# Some Contra-Indications for the Use of Opiates1Read before the Philadelphia County Medical Society, January 27, 1892.

**Published:** 1892-05

**Authors:** Marie B. Werner

**Affiliations:** 1010 Clinton Street; Philadelphia


### SOME CONTRA-INDICATIONS FOR THE USE OF OPIATES.[1]

By MARIE B. WERNER, M. D., Philadelphia.

My object in presenting this subject before the society tonight is manifold. I wish to show that in general practice the indications for the use of opiates are limited. We must fully realize that we have a broader basis for medical science than symptomatology, in order to give our patients the full benefit of our knowledge.

Our position being at all times one of trust, we must endeavor, in helping our patient, to find the cause and remove it, rather than hide the symptoms it gives rise to, by an opiate. Aside from the possible mental disturbances such a course of treatment might induce, it often materially complicates surgical efforts to relieve patients, the results of which we have repeatedly heard discussed at these meetings.

In order to discuss this drug with due fairness, it will be necessary to give a few moments to the consideration of its physiological action on the human economy. Prof. Wood, in his Treatise on Therapeutics, says:

> When opium is taken in such a dose as to produce its mildest physiological effects, it exerts a quieting influence, inducing a peculiar dreamy condition. After a length of time, varying according to the idiosyncrasies of the patient and the dose of the drug, this condition passes into sleep, either light, dreamful, natural, or heavy and deepening into stupor. On awakening, the patient may return at once to his normal condition; but very often he experiences a state of depression, shown by languor, a little headache, nausea, or even vomiting, which may last for some hours. One of the most common of these departures from the ordinary course of symptoms, is an excessive depression following the sleep produced by moderate doses of the medicine. This state is seen, so far as my experience goes, most usually in females of weak, nervous organization, such as are peculiarly liable to attacks of neuralgia. The symptoms are a feeling of weakness and prostration, often accompanied by chilliness, dull headache, and giddiness, but especially marked by intense nausea and frequent vomiting.

Bartholow states that, as a rule, opium does harm in all gastro-intestinal maladies in which there is a deficiency in the proper secretion, or a suspension of the functions of the liver and the kidneys.

Dr. J. B. Mattison, in his valuable paper read before this society in October, 1890, entitled The Renal Status of Opium Habitues,[2]

1. Read before the Philadelphia County Medical Society, January 27, 1892.
2. Buffalo Medical and Surgical Journal, Vol. XXX., p. 332.

arrived at the following conclusions, after a careful analysis: First—The habitual use of opium, in any form, will cause organic renal disease. Second—The changes most likely to be met with are cirrhotic. Third—The rationale is threefold,—vasomotor changes, impaired general nutrition, and inflammatory action, due to non-eliminated irritant products. I would further call attention to the valuable contributions of Dr. A. Haig, published in the *British Medical Journal*, 1890. In his studies on the influence of opium and morphia on uric acid, also of the retention of this latter product in the human economy and its relation to the causes of disease, his observations and experiments proved to him that the administration of opium or morphia caused retention of uric acid accompanied by a reduction of arterial tension; that when the effect passes off there is a rebound with excessive excretion of uric acid and marked high tension, often accompanied by headache and mental depression.

Let us look carefully into these statements and compare them with our practical experience; we find that after lulling pain and induction of sleep, we come to a period of depression, even after a moderate dose of the drug. This depression is usually followed by a certain loss of resistance to bear any renewal of pain, and, in consequence, it becomes necessary to repeat the use of the drug. Indeed, when we study carefully its action on a previously weak, nervous organization, we find that the description given by Prof. Wood very ably describes the case for us. He tells us: "The symptoms are a feeling of weakness, prostration, chilliness, dull headache, nausea, etc." In the face of this, the questions must certainly present themselves to us: Is it wise to simply gratify the desire of the patient? Would it not be better to study the cause and remove it, rather than hide the symptoms which lead us to the origin of the trouble? I refer to cases in everyday practice, cases in which a periodical monthly pain is lulled to sleep by several doses of morphia or opium, while the proper cause is entirely left out of sight. There is no doubt in my mind that many instances have come to all of us where a case of chronic constipation or continuous indigestion combined with a nervous, irritable temperament, perhaps added to that, or independent of this, an unpleasant skin eruption, etc., claimed our attention.

Quite often a question regarding any menstrual difficulties shows that there is some pain, often varying in severity, and the next question, What do you do for the pain? will often elicit the answer: Oh, I take a little paregoric, or, I have some pills or suppositories I use; perhaps previously given to some member of the family for pain. Not infrequently an investigation will show that the main ingredient is some opiate.

In our day of progressive medical science, it becomes necessary that we should join hands and forces, first to see if the proper hygienic rules regarding clothing, exercise, and cleanliness are scrupulously carried out, and that the proper functions of secretion and excretion are thoroughly understood. If, aside from these precautions, there is still pain, a local investigation should be made by the physician, the condition carefully studied, and the cause removed if possible. To give an opiate in such cases I consider criminal, since the patient receives a double injury; not only is she not relieved permanently, but she is robbed of much of her normal resistance, and I fear many have become chronic invalids; for it is a constant struggle to overcome the after-effects of the opiate before the time arrives for another relief of the same sort. If the patient escapes becoming addicted to the opium habit, she cannot escape the local gastro-intestinal irritation, which is invariably set up, and which will defy all medication so long as its cause remains.

I can at this moment call to mind three cases in which, three weeks out of four, all sorts of laxatives are used to overcome the amount of morphia taken during the fourth week. The complexion is sallow, the breath heavy, and the skin impure; and how can it be otherwise? If an examination reveals no functional trouble other than the local congestion, or possibly some displacement downward, induced by improper clothing or a lack of attention to the proper secretions, is it not at once clear that an opiate, in the long run, increases the cause of pain? Lulling pain induces no cure, and the resulting constipation acts in two ways to make the patient worse: (1) The pressure of a distended bowel. (2) The absorption of effete products. Let such conditions continue for some years, as they often do, and we have other factors entering the field to make life miserable,—sluggishly acting liver or kidneys, a wornout stomach, and not infrequently a nervous wreck.

In order to emphasize this point, I shall only have to call attention to a series of comparative experiments on animals[1] made by Dr. Edward Levinstein, and reported in his book on Morbid Craving for Morphia. His deductions from a number of experiments are as follows:

1. Covering a space of time from six days to five weeks.

1. That internal application of morphia sooner paralyses the digestive powers of the stomach than the subcutaneous injection.

2. Both ways of administering morphia bring on functional disorders of the secreting nerves.

3. Both cause catarrh of the stomach and intestinal tract.

4. Large doses of morphia given internally cause a subacute catarrh of the stomach, on account of the irritating chemical action of the morphia.

5. The subcutaneous injection of morphia causes a chronic catarrh of the intestines in a mechanical manner. In consequence of the impaired influence of the secreting glands, due to the action of the morphia, the secretion of the digestive fluids is stopped altogether, or at least diminished in quantity, and consequently the intestinal tract is encumbered for a longer time by the ingesta.

The same author speaks of amenorrhea and sterility as being a sequence to the continued use of morphia, drawing largely upon the results of his own observations, and accepts Pflueger's theory in explanation of it.

In these days, when "preventive medicine" is being advocated by all of us who desire to place medical science on the highest standard, should we not think many times more than twice before we write a prescription for an opiate to relieve pain? In the face of all these facts, it becomes a serious question of right and wrong if we stop short of exerting all our knowledge to study the *cause* of the pain we have been called upon to alleviate. Often the prescription book is entirely useless, unless its blanks could be filled with directions to the patient how to dress, eat, and give herself the physical care she needs.

The use of morphia after pelvic operations has been discarded by most of our operators, and clearly has been the means of reducing mortality rates, as well as of obviating many of the dreaded after-complications. I recall, in one of my early operations, the advice given by one of our older physicians, to rely on opium and calomel, which I followed, with the result of having on my hands a sufferer from insomnia and chronic constipation after I had discontinued its use. Had I not been careful to destroy all prescriptions, I feel certain I would have had more trouble. Another case comes to my mind, of a patient who had a section done for some pelvic trouble, by a physician who also believed in the opium after-treatment. This patient came under my care later, and confessed that she had often helped herself to suppositories after the doctor had stopped their use. My object in referring to these cases is to show the danger of setting up the morbid craving and its attendant evils, with only medicinal doses, and this in the space of two or three weeks, showing at once the danger a prescription containing an opiate may give rise to in the hands of a nervous patient who has periodical attacks of pain. Such cases do not always reach the state necessary to require hospital treatment, hence are often exceedingly vexing to the physician and surrounding friends. A direct accusation to the patient would often fail to bring the desired results, while the friends and relatives cannot always be relied upon for the tact and discretion so necessary. For that reason the physician must often exercise a vast amount of patience and time, to educate the ones immediately concerned and prove the deleterious effect the use of opiates has; and, *perhaps*, you can cure your patient!

It may, perhaps, be of interest to quote from a discussion on morphia in the British Gynecological Society, 1889. Dr. Bantock gave as his experience after surgical operations that patients were much better off without it,—they escaped the restlessness which was left as the opium wore off. Dr. Bedford Fenwick called attention to the fact that opium increased the congestion of the kidneys to a dangerous extent, and might even go to a complete suppression of urine; also, that it caused a complete atony or paralysis of the muscular tissue of the intestines, thus preventing their acting. Dr. R. T. Smith had given two doses of a quarter of a grain of morphia each in a case of severe shingles; the patient had suppression of urine for twenty-four hours. Dr. Thomas Savage, in his address read at the annual meeting of the Birmingham and Midland Counties branch of the British Medical Association, says:

> It is not long since it was the custom to administer opium and morphia as a routine treatment in all cases of peritonitis and any other conditions in the abdomen. We have now learnt the inadvisability of so doing. May we not extend the withholding of these and similar drugs in other states? I have myself thought that the general practitioners rely too much upon anodynes.

In this matter of too sympathetic and assiduous medical treatment, errors rather of judgment than intentional are often committed.

Of no less importance is the behavior of an opiate on a patient of uric acid diathesis, in which a demand for the relief of pain on the part of the patient often becomes urgent. Here again the researches of Dr. A. Haig shows us that the drug tends to store up the acid, that when elimination begins to take place there is often a return of the pain, the patient again demanding relief; in this manner a cycle can easily become established. These pictures teach us the importance of keeping the drug *entirely out of the reach of the patient*, and the necessity of its *careful* and *conscientious* use where it may be indicated.

1010 CLINTON STREET.

